# Community-Engaged Mental Health and Wellbeing Initiatives in Under-Resourced Settings: A Scoping Review of Primary Studies

**DOI:** 10.1177/21501319251332723

**Published:** 2025-04-21

**Authors:** Muhammad Chutiyami, Natalie Cutler, Sopin Sangon, Tusana Thaweekoon, Patcharin Nintachan, Wilai Napa, Phachongchit Kraithaworn, Jo River

**Affiliations:** 1 School of Nursing and Midwifery, University of Technology Sydney, Sydney, Australia; 2 Northern Sydney Local Health District, Sydney, Australia; 3 Ramathibodi School of Nursing, Faculty of Medicine Ramathibodi Hospital, Mahidol University, Bangkok, Thailand; 4 Faculty of Health, University of Technology Sydney, Sydney, Australia

**Keywords:** community engagement, initiatives, mental health, wellbeing, under-served

## Abstract

**Introduction:**

Community-engaged initiatives are identified as promising to improve the health of communities with limited resources. This review aims to examine community-engaged mental health/wellbeing initiatives across Low- and Middle-Income Countries (LMIC) and under-resourced settings of High-Income Countries (HIC).

**Methods:**

We searched CINAHL, Embase, MEDLINE, PsycINFO, and Scopus databases to identify eligible primary studies until August 2024. Studies conducted in English language, involving community members in the initiatives’ design or implementation and targeting 1 or more mental health/wellbeing outcomes, were included.

**Results:**

About 35 studies (n = 35) reporting 29 mental health/wellbeing initiatives across LMIC-(n = 24) and HIC-(n = 11) were included. Programmes with high community engagement, including community-led initiatives, consistently reported positive mental health and well-being outcomes, including reduced clinical symptoms and enhanced personal recovery and wellbeing. However, mixed outcomes on initiatives’ impact on quality of life and diagnosed mental health conditions were evident. Various challenges, including cultural barriers, were noted, as was a lack of involvement of people with lived experience of mental health challenges.

**Conclusion:**

Community-engaged mental health and wellbeing initiatives in under-resourced settings have shown the potential to improve mental health outcomes and well-being when actively involving community members. Future work should focus on scalable initiatives and active inclusion of people with lived experience of mental health challenges.

Review protocol registration at https://doi.org/10.17605/OSF.IO/367BK.

## Introduction

Socio-economic deprivation, geographical isolation, and unfavourable geopolitical and environmental events – including poverty, inequity, remote living, war, displacement, social marginalisation, and extreme weather events – are implicated in high rates of mental distress (eg, acute anxiety), diagnosed mental health conditions (eg, schizophrenia), and psychosocial disability (eg, chronic depression affecting activities of daily living),^1^ collectively referred to as mental health challenges. A disproportionate burden of mental health challenges and disability occur in Low- and Middle-Income Countries (LMICs),^2^ as well as under-resourced settings in high-income countries (HICs), including ethnic and cultural minorities, as well as rural and remote populations.^3-7^ In these settings, mental health services are frequently underfunded, understaffed, or unavailable, with geographically concentrated services in urban areas leaving large portions of the population without access to mental health care.^8-11^

Community engaged initiatives have emerged as a potential approach to promote mental health and well-being in under-resourced settings and are more likely to be sustained over time.^12^ Community-engaged approaches can enable community action for health in under-resourced settings, and include the development of supportive environments, coping skills, and provision of community-based care, as well as re-orienting health systems towards community needs.^13^ These approaches seek to actively involve communities in 1 or more aspects of the design or adaptation, implementation, and/or evaluation of mental health and wellbeing initiatives.^14^ There has also been an increasing push for meaningful engagement of people with lived experience of mental health challenges as a matter of respect, dignity, and justice, as well as a means of tackling discrimination and improving health.^15^

The ‘treatment gap’ – the proportion of people with mental health challenges who do not receive treatment or care – exceeds 85% in LMICs, compared to only 40% in HICs.^16,17^ However, in HICs, people in rural settings, low-income groups, and ethnic and cultural minorities are also less likely to receive care for mental health challenges compared to those in the general population.^18-22^ Digital health solutions are also often inadequate due to poor infrastructure, limited access to technology, or low literacy.^11^ Community engaged approaches have been proposed as a potential solution to address the mental health treatment gaps in under-resourced settings. Community-engaged approaches sit on a continuum of participation from low-level, community-oriented approaches, where community members are informed and called to join an initiative; to mid-level approaches, where community members are consulted in the design/adaptation and/or involved in the delivery or evaluation of an initiative; to high-level community-engaged approaches involving collaboration and decision-making with community members in the design, implementation, and/or evaluation, as well as community-led approaches, where community members lead in designing, implementing, and/or evaluating initiatives.^13^

High-level community engagement, particularly community-led initiatives, have the potential to promote mental health and well-being in under-resourced settings, and are more likely to be sustained over time.^12^ High-level engagement can leverage local knowledge, resources, and social networks to address mental health needs in contexts where formal healthcare systems are often inadequate or inaccessible,^23^ and offer a potential solution to bridge the gap between need and mental health care, by mobilising local resources and building on existing social structures.^13,14^ Implementation can take various forms, such as peer support groups, training of lay health workers for screening, referral and delivery of brief psychosocial interventions, and community-based psychosocial programmes.^24^ By engaging community members as active participants rather than passive recipients of care, community-engaged approaches can increase mental health services’ reach and cultural acceptability while fostering community resilience and social cohesion.^25^

Few comprehensive reviews currently address community-engaged responses to mental health challenges,^26,27^ with no review to our knowledge specifically investigating different levels (low, middle, high) of community-engaged approaches to mental health care in under-resourced settings. This scoping review aims to address this gap. It will map the existing literature on community-engaged mental health and wellbeing initiatives in under-resourced settings, targeting specific outcomes, including the level of community engagement, characteristics, impact of the programmes, and reported barriers/facilitators to implementation.

A scoping review is particularly appropriate for this topic as it allows for a broad exploration of the existing literature, identifying key concepts, gaps, and evidence across diverse contexts and approaches. The scoping review will further identify specific areas to be explored further in a systematic review or a primary study. In synthesising the evidence, we aim to support the identification of promising evidence-based community-engaged initiatives and areas for future investigation in this rapidly evolving field to inform policy, researchers, and practitioners working to improve mental health care access and outcomes in resource-limited contexts.

## Methods

### Protocol and Registration

We reported this review in line with the PRISMA extension for scoping reviews (PRISMA-ScR).^28^ We use a 6-stage scoping study methodological framework outlined by Arskey and O’Malley,^29^ incorporating recommendations of Levac et al.^30^ The final (optional) stage of the review (stakeholder consultations) is intended to be conducted as a separate follow-up study. The stakeholder consultations do not affect the interpretation of this review’s findings. The review protocol was registered at Open Science Framework (OSF) at https://doi.org/10.17605/OSF.IO/367BK.

### Eligibility Criteria

Published studies were included if they were community-engaged approaches that involved collaboration with communities in the design or implementation of the initiatives; targeted 1 or more mental health outcomes; and were conducted in under-resourced settings (LMIC, or HIC in settings with limited health resources); and reported in English. Primary quantitative, qualitative, and mixed-methods research studies including RCTs, cohort studies, pre-posttest designs, analytical cross-sectional studies, and qualitative interview/focus group studies were included. Studies were excluded if they were not community-engaged, did not include a mental health component, were opinion papers/secondary research studies and articles, or were in languages other than English.

### Information Sources

Five key databases were searched: CINAHL, Embase, MEDLINE, PsycINFO, and Scopus. An initial search was conducted in May 2023 to identify eligible studies with no restriction on the year of publication, and an update in August 2024 using the identical search strategy to identify new publications from May 2023 to August 2024. The reference lists of eligible studies were also searched to identify potential articles missed in the database search.

### Search

The search was conducted by 2 authors (JR and SS). The search process combines index and MeSH terms, as appropriate, to identify potentially relevant studies. The search terms were in line with the PICO framework (Population – LMIC and under-resourced HIC community settings; Intervention – mental health or wellbeing initiatives; Comparator – none; Outcomes; characteristics of initiatives and promotion of mental health and well-being). A search validation was conducted with one of the authors (JR) and an academic librarian to ensure that relevant studies (a number of known sources) were captured in search terms. Adjustments were made, and the final search terms, as detailed in Supplemental Table 1, were used to identify relevant studies.

### Selection of Studies

All the authors were involved in screening the search results. First, the database limiters/expanders, such as ‘Apply equivalent subjects’, were used to refine the search. Second, duplicates were removed through endnotes and Covidence. Third, the title/abstract/keywords of the potentially eligible studies were screened independently by the 2 teams of reviewers (University of Technology Sydney and Mahidol University) in line with the identified inclusion and exclusion criteria. Fourth, the full text of all potentially relevant articles was retrieved and screened independently by the same teams as above in line with the eligibility criteria.

### Data Charting Process

Data extraction was conducted by 4 reviewers (NC, SS, PK, and TT) in parallel using a Microsoft Excel extraction template designed by the review team. The first aspect of information extracted included study characteristics such as the research authors, study design, and participants’ characteristics. The second aspect of the extraction collected data related to the review aims, including mental health care initiatives, level of community engagement, the detail of intervention outcomes, and authors’ conclusions. Data on mental health outcomes were extracted based on clinical mental health as well as personal recovery indicators. Clinical mental health outcomes included data on reduction in mental health symptoms (eg, anxiety and depression), whereas personal recovery and wellbeing indicators extracted data on improvements in quality of life, resilience, social functioning, interpersonal relationships, and mental health awareness.

The data extraction was conducted in stages. First, the review team discussed the extraction process and outcome in meetings. As part of these meetings, a consensus was reached to categorise the methodology of the included studies in line with the JBI categorisation of research designs to ensure consistency. Second, a reliability verification of extraction by the 4 reviewers through a meeting was conducted following the extraction of 3 (10%) of the studies to ensure consistency. Third, the extraction of the remaining articles by the 4 reviewers was conducted.

### Critical Appraisal of Included Studies

Two reviewers (PN and WN) assessed the methodological quality of the included studies using the JBI critical appraisal tools for quantitative and qualitative studies^31^ and Mixed Methods Appraisal Tool version 2018 for mixed-methods studies.^32^ Differences between the 2 reviewers were resolved by discussion between the reviewers. Further disagreements were resolved through a meeting by the review team. An example of this disagreement is in the critical appraisal of the study by Jayaram et al^33^ in which the 2 reviewers perceived the methodological design as a mixed-method and case study, respectively. Following a meeting with the review team, the study by Jayaram et al^33^ was categorised as a case study.

### Synthesis of Results

Considering the heterogeneity in reporting of included studies (eg, methodology and context), a narrative approach was used to synthesise the data. This involved summarising the descriptive numerical data followed by a thematic analysis of the textual data. The synthesis considered the characteristics of the mental health or wellbeing initiatives, the country’s income level, and the population context as appropriate. The mental health or wellbeing initiatives (referred to as programmes) were categorised based on the level of community engagement.

#### Community Engagement

In line with the WHO continuum of participation in community-engaged approaches,^19^ we defined and categorised the following levels of community engagement for the purpose of this review;

##### Low-Level Community Engagement

Minimal or no participation of community members in the design or implementation of a mental health initiative. Community members’ role is characterised as passive – with little to no influence over the design or implementation phases. The communities are informed about a mental health initiative and are invited to participate in activities that have been pre-designed.

##### Mid-Level Community Engagement

Community members are consulted in the design or adaptation of a mental health initiative and may be involved in implementation. For example, they may have a say in shaping the content or assisting in the delivery of an initiative. However, the community members have minimal influence, and final decisions about the design and implementation of the initiative sit with external professionals or researchers.

##### High-Level Community Engagement

Substantial collaboration with community members in the design/adaptation and/or implementation of a mental health initiative. At this level, community members are partners with external professionals or researchers in shaping or delivering the initiative.

##### Community-Led Engagement

A subset of high-level of engagement where community members lead the design and/or implementation of mental health initiatives, which may or may not be supported by external professionals or researchers. This is also a high-level approach but is distinguished from collaboration as community leadership in 1 additional aspect.

#### Impact of Initiatives

The impact of community-engaged mental health and wellbeing initiatives was assessed using a comprehensive context that evaluates both clinical recovery and personal recovery and wellbeing outcomes. Clinical recovery focusses on improvements in clinical symptoms such as anxiety, depression. These outcomes were measured using validated scales such as the Depression Anxiety Stress Scale (DASS-21), Generalised Anxiety Disorder scale (GAD-7), Patient Health Questionnaire (PHQ), or General Health Questionnaire (GHQ). Measures of personal recovery and well-being emphasise improvements in quality of life, resilience, social functioning, interpersonal relationships, and mental health awareness. These outcomes were evaluated using validated tools such as the WHO Quality of Life-BREF (WHOQoL-BREF) or similar instruments. To ensure cultural and contextual relevance, self-developed tools tailored to the specific populations and settings of the initiatives were also included. This dual focus on clinical symptoms and broader well-being ensures a holistic understanding of the impact of community-engaged initiatives in diverse contexts.

## Results

### Selection of Included Studies

**Figure 1.**
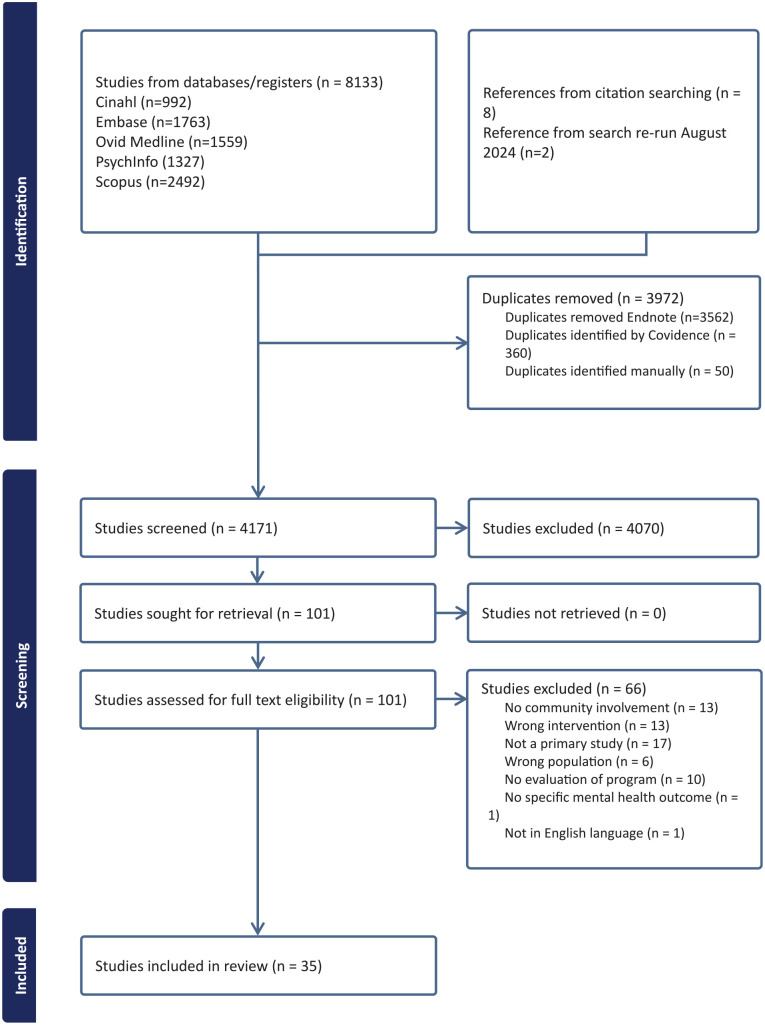
PRISMA flow chart indicating selection of included studies.

Following the initial search in 2023, we identified 8133 articles. A further 8 studies were identified through a citation search, and 2 additional studies were identified in the August 2024 search update. After duplicate removal, 4171 studies underwent title/abstract screening, and 4070 records were excluded for not meeting the eligibility criteria. The resulting full text of 101 potentially relevant articles were retrieved and screened in line with the eligibility criteria; 66 studies were excluded for reasons such as no community involvement (in design or implementation), wrong intervention (not a mental health initiative), wrong population (population from high-income countries without a clear indication of being under-resourced), and lack of programme evaluation (from target population or stakeholders involved, eg, community leaders and volunteers). The remaining 35 studies fully met the inclusion criteria (Figure 1).

### Characteristics of Included Studies

The 35 included studies comprise 29 various community-engaged mental health initiatives across high to low-income countries (Table 1). The majority of studies were conducted in LMICs, mainly in India (n = 10) and Ethiopia (n = 3). Studies in under-resourced populations in HICs were conducted in the USA (n = 5), Australia (n = 5), and England (n = 1). Of the 35 studies analysed, the majority used mixed methods (n = 12), and quantitative non-randomised controlled trials (n = 9) based on JBI categories of study designs.^31^

**Table 1.** Summary/Characteristics of Included Studies.

Authors	Country	Study aim	Community in which study is set	Characteristics of study participants	Participant numbers	Study design (JBI categorisation)
Ali et al^34^	USA	Assess how remote, peer-led intervention improves mental health	Church congregation in the Bronx, New York	African American congregants aged 18+.	n = 79 Genders not reported	Mixed method study
Anwar-McHenry et al^35^	Australia	Report impact of culturally adapted intervention	Aboriginal people in Roebourne community	Aboriginal community members. About 66% (21) were aged over 40.	n = 31 (F = 25, M = 0)	Cross-sectional studies/surveys
Appiah et al^36^	Ghana	Explore experiences, benefits, and recommendations to improve a positive psychology intervention	Population of 4 low-income communities in Ghana	People who had participated in the intervention. Aged 19-58 years. Mean age = 34 years	n = 18 (F = 10, M = 8)	Qualitative study
Arechiga et al^37^	Sierra Leone	Evaluate immediate and 6-month effectiveness of intervention post-Ebola.	Population of community in Sierra Leone	Paraprofessionals (nurses, teachers, business, and farming). Mean age = 33.7 years	n = 40 (F = 31, M = 9)	Non-RCT study
Asher et al^38^	Ethiopia	Describe the process used to design an acceptable and feasible intervention for local context	Population of Sodo district, rural Ethiopia	1. People with diagnosis of schizophrenia 2. Caregivers 3. Community leaders 4. Community health workers	n = 51 1. n = 5 (F = 2, M = 3) 2. n = 17 (F = 8, M = 9) 3. n = 7 (F = 0, M = 7) 4. n = 20 (no genders reported)	Qualitative study
Asher et al^39^	Ethiopia	Assess acceptability and feasibility of intervention for schizophrenia.	Population of Sodo district, rural Ethiopia	People with diagnosis of schizophrenia or related disorder, aged 19-60 years	n = 10 (F = 5, M = 5)	Mixed method study
Asher et al^40^	Ethiopia	Evaluate competence of lay health workers in delivering intervention for people diagnosed with schizophrenia	Population of Sodo district, rural Ethiopia	Lay health worker trainees. Aged 20-37 years. Mean age = 23 years	n = 10 (F = 5, M = 5)	Mixed method study
Balaji et al^41^	India	Describe the development of a lay health worker-led intervention for schizophrenia	Population of Goa state, and Satara and Kanchipuram districts	1. People with diagnosis of schizophrenia 2. Caregivers No age details reported	1. n = 32 2. n = 38 Genders not reported	Mixed method study
Brown et al^42^	England	Evaluate co-produced, community-led intervention for mothers	Population of socially deprived inner-city London borough	Mothers participating in the intervention. Aged 22-53 years. Mean age = 34 years	n = 61 (F = 61, M = 0)	Non-RCT study
Bryant et al^43^	USA	Describe the development of a faith-based intervention to improve mental health access	Population of Mississippi County, Arkansas	Pastors, parishioners, and African-American men with history of stress or depression. No age details reported	n = 24 (F = 0, M = 24) No details for separate cohorts	Qualitative study
Capp et al^44^	Australia	Develop and evaluate intervention to support suicide prevention	Population of Shoalhaven district, NSW	Aboriginal Population or employees of Shoalhaven Aboriginal Interagency Network. Aged 19-55 years, mean age = 36 years	n = 44 Aboriginal people (F = 40, M = 4)	Non-RCT study
Chibanda et al^45^	Zimbabwe	Assess acceptability and feasibility of intervention for women	Population of suburb Mbare in Harare	Experiencing depression and common mental health disorders	n = 320 (F = 224, M = 96)	Non-RCT study
Chomat et al^46^	Guatemala	Test acceptability, feasibility, and impact of intervention	Five rural Mam communities in San Juan Ostuncalco and 3 peri-urban K’iche’ communities in Quetzaltenango city	Pregnant or <2 years postpartum. Aged 15-44 years, mean age = 26 years	n = 84 (F = 84, M = 0)	Mixed method study
Chung et al^47^	USA	Examine collective efficacy, community engagement, and community partnerships in research process	African American community of South Los Angeles	Attendees at arts event intervention. Aged 18+ years. A 95% African American	n = 87 (F = 54, M = 33)	Cross-sectional studies/surveys
Giusto et al^48^	Kenya	Develop and explore the feasibility and acceptability of intervention	Population of Eldoret town in Rift Valley Province of Kenya	Fathers who experience problem drinking. Aged 30-48 years. Mean age = 39 years	n = 9 (F = 0, M = 9)	Qualitative study
Iheanacho et al^49^	Nigeria	Explore clergy-led intervention for depression	Population of Enuga state, Nigeria	1. . Catholic clergy. Mean age = 46 years 2. Women diagnosed with depression. Aged 19-65 years. Mean age = 42 years	1. n = 13 (F = 0, M = 13) 2. n = 28 (F = 28, M = 0)	Mixed method study
Jayaram et al^33^	India	Evaluate effectiveness of female village leaders in treating mental disorders	Community in villages near Mugalur in South India	Indigenous women. No age details reported	n = 2 (case studies). No individual details reported	Case series/case study
Joag et al^50^	India	Evaluate feasibility, acceptability, and cultural appropriateness of programme	Population of Nashik district in Maharashtra	1. Lay community members trained to deliver intervention (Champions). Aged 28-45 years 2. Population of suburbs: a. Peth (intervention) and b. Trymbak (control). Over 75% aged 18-39 years	1. n = 16 (F = 8, M = 8) 2. a. n = 829, b. n = 841 (Approx equal F & M)	Mixed method study
Kermode et al^51^	India	Explore mental health beliefs, issues, and programme impacts	Population of Maharasthtra, India	1. Volunteer health workers 2.Village women Aged 25-64 years. Mean age = 44years	1. n = 16 (F = 16, M = 0) 2. n = 16 (F = 16, M-0)	Qualitative study
Kidia et al^52^	Zimbabwe	Assess intervention implementation, acceptability, feasibility, and attitudes	Population of Zaka district in Zimbabwe	People involved with the intervention: 1. volunteer health workers, 2. nurses, 3. community leaders, 4. service users. Ages not reported	n = 32 (1 .n = 12, 2. n = 6, 3. n = 5, 4. n = 9) Genders not reported	Qualitative study
Lam et al^53^	USA	Evaluate and compare effectiveness of 2 different approaches for depression	Population of 2 Los Angelese communities: South Los Angeles and Hollywood Metro	People with diagnosis of depression utilising services. Aged 33-59 years. Mean age = 46 years	n = 1018 (F = 595, M = 423)	Non-RCT study
Lee et al^54^	Uganda	Evaluate impact of community-led theatre campaign on stigma	Population of Buyende District, Uganda	Community members who attended intervention (pre- and post-). No age details reported for pre-intervention. Post-intervention: Mean age = 38.7 years	1. Pre-intervention n = 101 (no gender details reported) 2. Post-intervention n = 57 (F = 44, M = 13).	Mixed method study
Lund et al^55^	Kenya	Evaluate mental health, economic, and quality of life outcomes post-intervention	Population of Meru South and Nyeri North districts of Kenya	People living with severe mental health or neurological disorders. Aged 18+ years. Mean age = 35 years	n = 203 (F = 104, M = 99)	Cohort study
Mathias et al^56^	India	Identify community features influencing outcomes	Population of Dehradun district in Uttarakhand	People with post-pandemic stress disorder, carers, community leaders, and community-based government functionaries. No age details reported	No details reported	Mixed method study
Mendel et al^57^	USA	Evaluate effectiveness of kickoff conference for community engagement	Population of 2 Los Angelese communities: South Los Angeles and Hollywood Metro	Conference attendees. No age details reported	n = 187 Genders not reported	Mixed method study
Nasir et al^58^	Australia	Evaluate cultural appropriateness and key components for effective suicide prevention training programme	Communities in southern Queensland with high suicide prevalence	Aboriginal and non-Aboriginal community members. No age details reported	No details reported	Qualitative study
Nguyen et al^59^	Vietnam	Evaluate intervention’s acceptability, effectiveness, and impact	Population of Tien Ngoai commune in Ha Nam province	People diagnosed with serious mental illness who attended the intervention. Aged 25-66 years. Mean age = 45 years	n = 68 (F = 43, M = 25)	Mixed method study
Nickels et al^60^	El Salvador	Explore participant perceptions of effectiveness and satisfaction with programme	Population of capital city San Salvador	1. People diagnosed with serious mental illness who attended intervention. Aged 21-45 years. Mean age = 37 years 2. Carers. Aged 26-62 years. Mean age = 37 years 3. Healthcare workers. Aged 37-53 years. Mean age = 46 years	1. n = 3 (F = 1, M = 2) 2. n = 4 (F = 4,M = 0) 3. n = 3 (F = 1, M = 2)	Mixed method study
Pathare et al^61^	India	Evaluate intervention’s impact on depression, anxiety, and functioning improvement	Population of Mehsana district of Gujarat	People with symptoms of depression and anxiety. Mean age = 45 years	1. Intervention group: n = 608 (F = 367, M = 241) 2. Control group: n = 583 (F = 334, M = 249)	Cross-sectional studies/surveys
Raguram et al^62^	India	Assess impact of healing temple stay on mental illness symptoms	Population of Velayuthampalayampudur, Dindugal district, Tamil Nadu	Community members with serious mental health disorders who stayed at the temple. No age details reported	n = 31 (F = 10, M = 21)	Non-RCT study
Shidhaye et al^63^	India	Evaluate effectiveness of intervention	Population of 30 villages in Amravati district, Vidarbha	Community members. Aged 18-56+ years), comprising: 1. With depression 2. Without depression	n = 1887 (F = 890, M = 997), comprising: 1. n = 213 2. n = 1674	Non-RCT study
Shields-Zeeman et al^64^	India	Describe intervention implementation and training of community volunteer champions	Population of state of Maharashtra	Community members trained to deliver identified intervention. No age details reported	1. Champions (n = 59) 2. Mitras (n = 264) Genders not reported	Case series/case study
Sun et al^65^	Australia	Investigate impact of intervention on social, emotional well-being of target Population	Aboriginal and Torres Strait Islander peoples from the State of Queensland	Aboriginal and Torres Strait Islander peoples who participated in the intervention. Aged 18+	n = 117 Genders not reported	Non-RCT study
Taylor et al^66^	Australia	Evaluate effectiveness of intervention programme	The community of people who are involved in running The Station programme	The Station members, volunteers, management committee, and staff. No age details reported	n = 25 Genders not reported	Qualitative study
van Ginneken et al^67^	India	Compare primary health worker-led models and assess scaling-up potential	Public and private not-for-profit primary and community mental health services in India	Staff involved in delivery of mental healthcare services. No age details reported	n = 246, comprising: 1. Primary health workers (n = 134) 2. coordinators (n = 33) 3. specialists (n = 40) 4. leaders (n = 34) 5. other staff (n = 5) Genders not reported	Qualitative study

Most studies focussed on preparing community members to deliver training, surveillance, and/or basic mental health interventions (eg, gatekeeping for referral) for other community members (eg, Asher et al^40^). Some studies (eg, Brown et al^42^) were focussed on evaluating the impact of such interventions/programmes in terms of clinical recovery, which is defined as a reduction of clinical mental health symptoms and distinguished from ‘personal recovery’, which can occur with or without reduction in clinical symptoms, and is associated with improvements in social connectedness, engagement in meaningful social roles, hope and optimism for the future, rebuilding a sense of self, and empowerment^68^ Other studies focussed on well-being, or outcomes consistent with personal recovery (eg, Anwar-McHenry et al^35^ and Nickels et al^60^), or indicators of acceptability, feasibility, and cost-effectiveness (eg, Sun et al^65^).

The quality appraisal results of the included studies varied based on the research designs. Two^43,56^ of the 35 included studies received below-average quality ratings in line with the JBI tools. The 2 studies with below-average quality scores were included in the analysis to provide a comprehensive overview, and are acknowledged in the results section for the purpose of transparency. Details of the quality ratings are presented in Supplemental Table 2.

## Findings of the Review

### Community Engagement

The extent of community engagement varied across the 35 studies reviewed, which represented 29 mental health initiatives. The majority of the initiatives involved mid-level (n = 11) community engagement. Others were low-level (n = 2), or high-level (n = 9) community engagement without a community leadership role. Ten initiatives were community-led, with the leadership role in the delivery/implementation of the programme but not in the design (Table 2). The programme developed by Van Ginneken et al,^67^ which integrates 72 case studies in India, involved low-, mid-, and high-level community engagement. Additionally, the Gatekeeper Training Programme for Indigenous Australians was initially developed with mid-level community engagement^44^ but later evolved to community-led engagement to enhance cultural appropriateness and relevance.^58^

**Table 2.** Programmes, Characteristics, and Impact.

Programmes/initiatives	Reference	Target outcome (s) as described	Community engagement				Point of care			Setting			Programme impact on outcome (s)			Key comments
			Led	High	Medium	Low	Home	Community	Institution/ others	HIC	MIC	LIC	Positive	No difference	Unclear/ NA	
Act-Belong-Commit mental health promotion campaign	Anwar-McHenry et al^35^	Mental health promotion		✓				✓		✓			✓			Consultations with elders, community organisations and community members. Engagement with Aboriginal consultant and researcher. Following the campaign, 81% reported self-care, 74% family care, and 48% community care (social and emotional well-being) among Aboriginal adult community members.
Atmiyata programme	Pathare et al^61^	CMD and QoL	✓					✓			✓		✓			Community champions (trained volunteers) deliver the intervention. Significant recovery from CMD (anxiety and depression symptoms); Adjusted OR 2.2 (95% CI: 1.2-4.6) at 3 months and sustained at 8-month follow-up; Adjusted OR 3.0 (95% CI: 1.6-5.9).
	Joag et al^50^	Emotional stress and mental health disorders	✓					✓			✓		✓			Community champions (trained volunteers) were effective in accurately identifying 65% of cases of CMD, referrals of cases of SMD, counselling, and supporting people with mental disorders/distress. Reduction in GHQ among intervention vs controls; 43% vs 29%.
	Shields‑Zeeman et al^64^	Emotional stress and mental health disorders	✓					✓			✓				✓	The programme aimed to develop community-based mental health and social care pathways to reduce the treatment gap and contribute to the achievement of a higher quality of life for people with CMDs and SMDs.
Basic Needs’ Mental Health and Development Programme	Lund et al^55^	Mental health and development			✓		✓	✓	✓		✓		✓			Community involved in identifying individuals who could benefit from the programme. Baseline to 2 years; significant improvements in GHQ-12 (21.5[95% CI: 20.2-22.8] to 6[95% CI: 4.8-7.2]), GAF (78 [95% CI: 75.5-80.3] to 94 [95% CI: 90.7-97.3]), QoL (39.5 [95% CI: 38.6-40.4] to57.2[95% CI: 56.2-58.3]).
Collaborative Community Based Care (CCBC)	Balaji et al^41^	Schizophrenia			✓		✓				✓				✓	Trained lay health workers deliver the intervention under specialist supervision. Intervention developed from literature review. Successful development of community-based intervention; an acceptable and feasible intervention for treating schizophrenia in India.
CCM-SCM-TIRM	van Ginneken et al^67^	Mental health (depression, anxiety, and comorbidities)		✓	✓	✓	✓	✓	✓		✓		✓			Three categories of models were derived from a combination of 72 mental healthcare programmes (case studies) involving low to high community engagement in India. All models have shown a positive impact on mental health issues.
Community Resiliency Model (CRM)	Arechiga et al^37^	PTSD, Depression and Anxiety symptoms, Traumatic distress, and Resilience			✓				✓			✓	✓			Intervention developed by researchers. Participants were selected by community leaders. Administered in a community hospital. Improvement from the pretest to post-test in PTSD, Depression and Anxiety symptoms, and Traumatic distress (*P* < .05). Increased resiliency 6 months post-intervention.
CEP (Community coalition model)	Lam et al^53^	Depression		✓					✓	✓				✓		Community-based partners and members involved in the intervention design. Community and academic partner co-leaders had equal authority in the research. Administered in community agencies, for example, PHC. No difference in primary outcomes (Mental health QoL and PHQ-9 scores), but it increased the participation of eligible staff in depression training.
Community mental health project	Mathias et al^56^	Post Pandemic Stress Disorder (PPSD)	✓					✓			✓		✓			Led programme delivery. In each community, 5 community-based team members (employed staff) worked with community volunteers to build community knowledge, safe social spaces, and partnerships for action. Overall increase in community knowledge on mental health and social inclusion for PPSD and their families, and engaging partnerships for action, such as women careers
Community singing programme	Sun et al^65^	Social and emotional wellbeing		✓			✓	✓		✓			✓			Aboriginal leaders played central role in recruitment, study design, and implementation. The intervention was conducted and coordinated by local Aboriginal Community Health Service representatives. Community leaders and representatives were involved in organising the intervention. Significant differences were observed between the pre-and post-intervention phases for stressors like the death of family members.
Comprehensive Rural Health Project (CRHP)	Kermode et al^51^	Depression, suicide, and violence in women		✓					✓		✓		✓			Researchers developed interview themes, but local staff reviewed the interview themes. Interviewers were locally recruited and trained. Delivered in PHC centre. Programme activities were perceived to be effectively addressing the determinants of mental health, including stigma and discrimination.
Family Education, Support, and Empowerment Programme (FESEP)	Nickels et al^60^	Mental illness			✓			✓			✓		✓			Target community members participate as board members or as part of the coordinating Team but do not lead support groups. Programme had multiple benefits across social, functional, and economic dimensions and various achievements at organisational and national levels.
Friendship Bench	Kidia et al^52^	CMD			✓		✓		✓		✓		✓			Lay workers were trained and supervised by nurses to deliver the intervention. Delivered in rural areas. The intervention was acceptable but neither feasible nor sustainable.
	Chibanda et al^45^	Depression and CMD			✓		✓		✓		✓		✓			Lay workers were trained and supervised by nurses to deliver the intervention in communities with high HIV prevalence. Reduction in psychological symptoms and improved mental well-being among individuals.
Gatekeeper Training Programme (GTP) and modified programme though INSIST project	Capp et al^44^	Suicide prevention			✓			✓		✓					✓	Consultation with Aboriginal people on the content of the training programme. GTP for Australian Indigenous communities increases participants’ knowledge about suicide, greater confidence in the identification of people who are suicidal, and high levels of intention to provide help.
	Nasir et al^58^	Suicide prevention	✓					✓		✓					✓	INSIST project. Led programme delivery. Involvement of Indigenous researchers and participation of Indigenous community members and organisations. Culturally appropriate GTP requires the use of local language, culturally relevant content, generalisability to other Indigenous communities and the need for long-term sustainability.
Healthy Beginning Initiative (HBI)	Iheanacho et al^49^	Depression among women			✓				✓		✓				✓	Church leaders (clergy) were involved and were willing to be trained to enhance their capacity to provide mental health therapy for women. Administered in church. Women with diagnosed depression showed an overwhelming preference for clergy-delivered interventions for mental disorders.
Spirituality-Based programme	Ali et al^34^	Mental health and wellbeing		✓			✓	✓		✓			✓			Congregation leaders selected peer educators who delivered the programme. Delivered online. Reduced odds of depression, increased sense of community, social support, role of religion in health, and reduced trouble sleeping.
Inspired Life Programme (ILP)	Appiah et al^36^	Depressive symptoms and positive mental health				✓	✓	✓			✓		✓			All sessions are facilitated by trained psychology graduates. Positive experiences of the programme through self-reflection, practicality and relatability, mutual engagement and self-disclosure, and a sense of responsibility and accountability.
Community Partners in Care (CPIC)	Mendel et al^57^	Depression		✓				✓		✓			✓			Used collective efficacy and a community-of-practice among community stakeholders interested in addressing a community health need. Kickoff conference was effective in stimulating a collective sense of connection and efficacy to address depression/depression care in the community.
LEAD (Learn, Engage, Act, Dedicate)	Giusto et al^48^	Men alcohol use, depression, and family engagement			✓			✓			✓		✓			Community members were involved in the refinement and piloting of the intervention. Most participants reported that the programme helped them to reduce alcohol use, improve their mood and improve interactions at home.
Maanasi Clinic (meaning ‘of sound mind or strong-minded woman’)	Jayaram et al^33^	Women depression, Men alcohol abuse, and general mental health			✓		✓		✓		✓		✓			Involves training local women as CHWs with ongoing supervision and collaboration with primary healthcare. Positive attitude towards mental illness. Perceived CHW’s concern, compassion, and empathy towards patients’ illnesses and well-being. People with mental health challenges who accessed the programme felt empowered and assisted in self-employment.
Parents and Communities Together (PACT)	Brown et al^42^	maternal anxiety, depression, health literacy, and social support	✓					✓	✓	✓			✓			Led programme delivery. Co-production with community women who had participated in the pilot study. Significant overall decrease in mean GAD-7 (6.87 ± 5.6-4.76 ± 3.85) and PHQ-9 (7.66 ± 6.37-4.83 ± 4.15) scores.
Participant informed, psycho-educational, community-based intervention.	Nguyen et al^59^	Severe mental illness (psychotic symptoms)	✓					✓			✓			✓		Led programme delivery. Lay health workers and para-professional health workers delivered the intervention. No significant improvement in 6 common psychotic symptoms (mean difference = −1.26; 95% CI: 2.78, 0.25), but intervention had significant impact on personal functioning (mean difference = 5.91; 95% CI: 0.29, 11.53)
RISE CBR intervention	Asher et al^38^	Schizophrenia			✓		✓					✓			✓	Focus on CBR development. Participants (Stakeholders) perceived CBR to be acceptable and useful for addressing experiences of family conflict, difficulty participating in work and community life, and stigma.
	Asher et al^39^	Schizophrenia			✓		✓					✓	✓			CRB workers (lay people) trained to deliver intervention with supervision. Intervention may have a positive impact on functioning through the pathways of enhanced family support, improved access to health care, increased income, and improved self-esteem.
	Asher et al^40^	Schizophrenia and other mental health			✓		✓	✓				✓	✓			CRB workers (lay people) trained to deliver intervention with supervision. Improvement in CBR worker competence to assist patients with schizophrenia and their families
Talking Wellness (Arts Events)	Chung et al^47^	Depression		✓				✓		✓			✓			Community members collaborated in the design and delivery of the intervention. Programme was assessed during a film festival. Improved community engagement in depression care.
Temple Healing	Raguram et al^62^	Serious mental disorder	✓						✓		✓		✓			Led programme delivery. Temple leaders implement the programme as part of their daily routines. Individuals seeking help and their caregivers provided information, shared their experiences, and cooperated with the researchers. Administered in the temple. Improvement in SMD symptoms.
The Station Community Mental Health Centre	Taylor et al^66^	Mental health support and recovery		✓					✓	✓			✓			Collaboration between community and health service. Delivered at ‘The Station’ Community Mental Health Centre. The programme has the potential to improve well-being, enhance social support, empowerment, self-efficacy, and reduce stigma.
Theater play	Lee et al^54^	Mental illness stigma	✓					✓			✓		✓			Led programme delivery. Community collaborated in the design and implementation of the intervention. Decrease in stigma ratings. Positive changes in participants’ beliefs and attitudes towards mental health challenges and the importance of seeking help.
Trinity Life Management	Bryant et al^43^	Health disparities in stress, distress, and depression	✓						✓	✓					✓	Led programme delivery. To be led by community/ laypeople. The intervention was developed with community members who were part of a community advisory board. Administered in church. Involvement in programme positively changed some members’ attitudes and knowledge of health and research.
VIdarbha Stress and Health Programme (VISHRAM)	Shidhaye et al^63^	Depression	✓				✓		✓		✓		✓			Led programme delivery. Community-based health workers and non-specialist counsellors delivered interventions, including referrals to facilities. Programme was effective in detecting depression and intention to seek care.
Women’s Circle	Chomat et al^46^	Maternal distress and wellbeing	✓				✓	✓			✓		✓			Led programme delivery. Intervention was co-designed and facilitated by community representatives and leaders. Wellbeing scores and self-care/self-efficacy scores were higher among the intervention group than the controls.

NA: Not available. CCM-SCM-TIRM: Collaborative Care Models – Specialist Community Model – Training and Identification/Referral Models; CBR: Community Based Rehabilitation; CHW: Community Health Workers; CEP: Community Engagement and Planning; CMD: Common Mental Disorders; GAD: Generalised Anxiety Disorder; GAF: Global Assessment of Functioning; GHQ: General Health Questionnaire; INSIST: Indigenous Network Suicide Intervention Skills Training; PHQ: Patient Health Questionnaire; PPSD: Post Pandemic Stress Disorder; PTSD: Post Traumatic Stress Disorder; QoL: Quality of Life; RISE: Rehabilitation Intervention for people with Schizophrenia in Ethiopia; SMD: Severe Mental Disorders.

Programmes with high-level collaboration, some of which were community-led, were consistently perceived to have a positive impact among involved community members, for example, volunteers, leaders.^34,43,50,53,54,57,58^ Impact was also perceived positively by participants in the initiative or intervention, who are from the broader target communities.^33,34,35,37,42,45,46,47,50,51,54,56,59,61-63,65^ The use of community or social elements, such as community art events,^47^ collaboration with established community organisations like Aboriginal Community Controlled Health Services,^65^ or the involvement of community volunteers,^50,61^ community health workers,^63^ and religious leaders (below-average quality study),^43^ was found to be instrumental in encouraging active community participation.

Some programmes with low- to mid-level community engagement were also positively perceived by communities.^36,38,39,48,49,55^ A potential to translate some programmes into impactful interventions for the target communities was reported.^40,41,44^ Programmes such as the Community Resiliency Model, were designed by researchers or professionals, with community involvement limited to the management or implementation phases.^33,37,48,49,60^

### Types of Community-Engaged Programmes

#### Peer Initiatives

About 11 out of the 29 mental health initiatives primarily incorporated peers within the target communities as the basis for the intervention.^34,38-42,44-46,49,50,52,54,58,61,62,64^ The majority of these programmes were implemented in LMICs (Table 2). The peer support programmes were typically facilitated by non-professionals, such as community laypeople,^40,41,45,52,61^ religious/spiritual leaders,^34,49,62^ Indigenous people,^44,46,58^ or mothers or pregnant women.^42,46^ However, none of the included studies indicated that people with lived experience of mental health challenges were involved as peers.

In these peer programmes, laypeople were trained or supervised to deliver mental health screening and basic mental health or psychosocial interventions within their communities,^40,41.45,61,64^ and 1 study reported that aboriginal people were specifically trained to detect and respond to suicide risk among their peers.^44,58^ Raghuram et al^62^ reported on a unique temple healing programme where individuals diagnosed with mental health challenges and their families resided at a community temple free of charge, engaging in various temple routines, such as watering plants, to facilitate healing.

#### Collaborative Initiatives

About 14 out of the 29 programmes primarily involved collaboration between community members and health systems, healthcare providers, and/or researchers^33,36,37,43,47,51,53,56,57,59,60,63,65,67^ across both LMIC and under-resourced HIC settings (Table 2). In LMIC settings for example, Jayaram et al^33^ found that integrating women who were village leaders and community health outreach workers to identify and treat mental health conditions in an Indian rural community was both effective and acceptable. Mathias et al^56^ (below-average quality study) highlighted the potential of engaging different community groups, such as leaders, and preschool workers, to support families with mental health following pandemics. VISHRAM (Vidarbha Stress and Health Programme) involve lay community workers, who refer complex cases to primary care doctors.^63^

In HIC, Sun et al^65^ reported on a community singing programme for Aboriginal people in Australia, which involved collaboration between local Aboriginal communities and representatives from local Aboriginal Community Controlled Health Services. Chung et al^47^ examined a community art event targeting depression among African American communities in Los Angeles, which included spoken word sessions and photography exhibits, was implemented through collaboration between academic researchers and African American people. Other collaborative care initiatives with academics included the Community Resiliency Programme and the Trinity Life Management programme, which were developed by researchers but implemented by community leaders.^37,43,51^

#### Integrated Service Delivery

About 4 of the 29 programmes primarily integrated mental health services with other community services,^35,42,48,55,66^ primarily in HIC (Table 2). Among these, the Parents and Communities Together (PACT) initiative involves peer support in addition to the integrated service delivery nature of the programme.^42^

These integrated service delivery programmes not only addressed mental health but also incorporated broader social and physical health initiatives, such as promoting well-being through community campaigns,^35^ social connectedness,^66^ family engagement,^48^ and overall quality of life.^55^ For instance, Brown et al^42^ reported that the PACT programme combined maternal literacy, social support, and mental health components. Lund et al^55^ also evaluated a programme in Kenya that integrated mental health care with poverty alleviation components, finding it to be both feasible and beneficial.

In these integrated service delivery programmes, implementation was typically conducted by trained lay community members acting as counsellors,^48^ or peers who were Indigenous persons,^35^ or mothers,^42^ or by a diverse team of stakeholders, including programme volunteers and management committee members.^66^

### Impact of Community-Engaged Initiatives

#### Improvements in Clinical Symptoms (Clinical Recovery)

Ten of the 35 studies reported improvements in clinical recovery (mental health symptom reduction). The community-engaged programmes promoted clinical recovery, including overall mental health symptoms assessed with Generalised Anxiety Disorder, Patient Health Questionnaire or General Health Questionnaire scales.^42,50,55,61^ Specifically, the initiatives showed efficacy in reducing symptoms or odds of depression,^34,37,42,45,47,48,50,61,62^ as well as reductions in anxiety symptoms,^37,42,50,61^ PTSD symptoms,^37^ and mental distress.^37,50^

The impact of initiatives on the symptoms of people diagnosed with mental health conditions such as schizophrenia revealed mixed results. Raguram et al^62^ demonstrated significant positive outcomes for clinical symptoms, with ‘thinking disturbance’, scores on the Brief Psychiatric Rating Scale improving from 12.45 (SD = 3.21) at initial assessment to 9.81 (SD = 4.42) at discharge (*t* = 3.701). Additionally, Lund et al^55^ highlighted significant improvements among individuals diagnosed with schizophrenia and bipolar disorders, with General Health Questionnaire (GHQ-12) scores improving from 21.5 (95% CI: 20.2–22.8) at baseline to 6.0 (95% CI: 4.8–7.2) after 2 years, reflecting substantial positive changes in overall health and well-being. However, Nguyen et al.54 reported no significant changes in clinical symptoms in their study of a support group for people with a diagnosed mental health condition. Other studies reported outcomes on individuals diagnosed with schizophrenia but did not assess the impact on the symptoms.^38-41^ Further information on Table 2.

#### Improvement in Well-Being, Personal Recovery, and Other Associated Mental Health Outcomes

About 29 of the 35 included studies reported improvements in well-being, including those consistent with personal recovery indicators (Table 2). High-level community-engaged initiatives, such as a singing programme, were effective in reducing stressors associated with life events, including the loss of family members or employment.^65^ Additionally, these programmes were associated with a reduction in mental health stigma and discrimination,^38,47,51,54,59,60^ as well as impacting positively on caste, gender, and disabilities stigma.^51^

Overall community-engaged programmes were found to promote overall well-being,^33-36,45,46,51,55^ with specific improvements linked to poverty alleviation,^55^ and improved mental health awareness.^35^ Other reported improvements included enhanced self-care,^35,46^ total satisfaction,^42^ increased self-esteem, and self-acceptance of mental health challenges.^39,60^ However, reported impact on health-related quality of life (QoL) varied considerably, with some studies finding positive results,^55,60^ and others reporting no significant differences between experimental and control groups.^53,61^ For example, Lam et al^53^ found no significant difference in mental health-related QoL when comparing groups above and below the poverty line in a community coalition model randomised trial. In contrast, Lund et al^55^ reported a significant improvement in mental health-related QoL scores, increasing from 9.7 (95% CI: 9.5-10.0) at baseline to 13.9 (95% CI: 14.1-14.7) after 2 years in a Basic Needs’ Mental Health and Development Programme, which was assessed using a single-group cohort design.

Initiatives also demonstrated potential in promoting social connection and participation,^61,66^ improving interactions and peace at home or within the community^48,65^ – including improving intimate relationships,^42^ strengthening social well-being and community relationships,^35,36,42,50,51,60^ and increasing family support and functioning.^39,50,59,60,62^ A programme aimed at empowering women led to greater self-determination,^51^ and a sense of community was strengthened through some interventions.^34,57^

Initiatives were also linked to increased income and economic stability,^39,48,51,55,59,60^ with some facilitating employment or self-employment opportunities for service users.^33,50,55^ Productivity, goal attainment, responsibility, and accountability improvements were also noted.^36^ The resilience of participants and their families, along with support in applying for social benefits, were additional positive outcomes of community-engaged initiatives.^50^

Other associated mental health improvements include increased access to mental health services,^39,50,54,57,63^ and improved help-seeking behaviours,^67^ and enhanced health literacy.^42,47,56,59,60,63,67^ Reduced mental health treatment delays,^67^ referral of cases of severe mental health challenges such as psychosis^50^ and enhanced caregiver competence to assist individuals with diagnoses of schizophrenia and their families^40^ were also reported. Collaborative efforts between specialists and community healthcare teams could provide long-term support for individuals with complex mental health challenges.^67^ One study also indicated increased advocacy for individuals with diagnosed mental health challenges,^60^ and 1 study found that engagement of community health workers resulted in positive attitudes in the community towards individuals with mental health challenges.^54^

### Summary of Impact of Initiatives Based on Level of Community Engagement

Of the 29 initiatives, low-engagement programmes showed positive participant experiences without significant clinical mental health symptom improvements. These programmes require additional efforts to ensure cultural relevance/feasibility. Medium-level engagement programmes involving community consultation, reduced clinical mental health symptoms (eg, Friendship Bench Programme). High-level engagement programmes, characterised by substantial collaboration with communities, demonstrated significant improvement in clinical mental health symptoms and personal-recovery indicators such as enhanced social inclusion (eg, Talking Wellness initiative). Community-led programmes delivered the most culturally relevant and sustained impacts, with initiatives like the Women’s Circle significantly improving wellbeing, although mental health symptom reduction was variable.

### Barriers and Facilitators to Implementation of Programmes

#### Perceived Acceptability, Availability, and Accessibility Barriers

Six studies highlighted common operational difficulties, including acceptability, availability, and accessibility barriers encountered during the implementation of community-engaged initiatives.^40,43,50-52,58^

Engagement with Gatekeeper programme among the Australian Indigenous population was adversely affected by the lengthy and perceived irrelevance of training activities, a barrier to acceptability.^58^ Additionally, primary healthcare doctors participating in the Atmiyata programme in India were reluctant to prescribe psychotropic medications due to insufficient training despite the availability of these medicines.^50^ Translating theoretical concepts into practical applications, particularly when training laypeople to implement a Rehabilitation Intervention for people diagnosed with schizophrenia in Ethiopia, was also perceived as problematic.^40^

Accessibility was another key issue identified by both service users and Village Health Workers (VHWs) in the Friendship Bench programme in Zimbabwe, which included difficulty in the transport of service users and poor remuneration of VHWs.^52^ The physical distance between the research team and the community setting in the Trinity Life Management programme hindered the establishment of solid rapport and active relationships with community members, which are essential for effective programme delivery (below-average quality study).^43^ One study also emphasised the critical need for reliable referral systems for individuals with complex mental health needs who cannot be adequately managed within primary healthcare centres or communities.^51^

#### Cultural and Contextual Barriers

Four studies detailed the linguistic and cultural challenges and necessary adaptations required for implementing initiatives in diverse settings.^39,43,54,63^

One significant barrier was the high responsibility placed on key community partners, such as pastors, which could strain their capacity to contribute effectively to programme development (below-average quality study).^43^ Additionally, translating mental health concepts into local languages proved challenging, requiring careful consideration to ensure cultural relevance and understanding.^54^ Some community religious groups were protective of their resources and reluctant to share assets, which hindered collaborative efforts (below-average quality study).^43^

In Ethiopia, community workers faced difficulties in accepting the autonomy and choices of individuals diagnosed with schizophrenia in the context of the community rehabilitation programme.^39^ Similarly, stigma against people diagnosed with depression led to a necessary shift in the focus of a programme towards stress management to better align with community perceptions.^43^ Stigma was also a reported barrier in other studies. For example, participants in 1 programme expressed reluctance to socialise with or live near individuals diagnosed with depression, highlighting how stigma can undermine the effectiveness of mental health initiatives.^63^

#### Facilitators/Enablers of Success

Thirteen studies identified key factors that contributed to the successful development or implementation of the community-engaged initiatives.^33,35,36,43,44,46,49,50-52,57,58,64^

Engaging local community people as partners in programme development^35,46,57^ and conducting active consultations at the programme’s inception^35,57^ were found to be crucial in promoting cultural safety and community acceptance. In an Indigenous suicide prevention programme in Australia, the need to train Aboriginal people to provide culturally safe care or screen for suicidality was identified as crucial for programme success^44^ and the involvement of the community as programme drivers enhanced the potential for sustainability.^58^

Building community trust^35,43,50,54^ and collaborating with key community figures, such as leaders,^35,36,43^ were identified as essential elements for ensuring programme success. The use of community health workers (CHWs) has shown potential for dispelling myths/misconceptions associated with mental health disorders, thereby reducing stigma.^33^

Context and language was also vital. The supportive and non-threatening environment of a temple, even in the absence of specific healing rituals, played a significant role in reducing clinical symptoms.^62^ VHWs in the Friendship Bench programme advocated for the need for implementation near their homes, and to be provided with bicycles to ease transport difficulties.^52^ Employing community-acceptable terms, such as ‘distress’ and ‘well-being’ instead of ‘depression’ and ‘anxiety’, also facilitated greater acceptance of programmes.^50^

Kermode et al^51^ emphasised the importance of addressing gender and power imbalances within communities and strengthening health systems to support these efforts. In the Atmiyata programme, community volunteers played a vital role in addressing gender and caste barriers, such as having women volunteers assist male service users, which mitigated some of the deeply rooted social divisions in Indian rural communities.^50^ facilitating the success of the programme. Additionally, the willingness of community volunteers to participate without financial compensation, and maintain frequent communication with district psychiatrists, were critical in securing professional consultations and identifying more effective treatment options for those in need.^64^ Iheanacho et al^49^ highlighted the need for programmes to incorporate culturally relevant approaches that recognise diverse explanatory frameworks for mental distress, including supernatural causes. Including physical health promotion alongside mental health interventions was also recommended to ensure comprehensive care.^36^

### Comparison Between Under-Resourced HICs and LMICs

Overall, the 29 mental health/wellbeing initiatives across LMIC and HIC country settings share both similarities and differences. In both settings, community engagement involved community members in the design or delivery of the intervention, with initiatives such as the Atmiyata programme^50,61,64^ (LMIC) and the Act-Belong-Commit campaign^35^ (HIC) leveraging community involvement to improve access to mental health care, fostering social inclusion, and reduce stigma. Programmes in HICs often incorporated formal collaborations (structured and organised roles and processes) among community organisations, healthcare providers, and academic institutions, as seen in the Community Partners in Care initiative,^57^ which used a community-of-practice model to enhance collective efficacy. LMIC programmes also collaborated beyond the community. For example, the Basic Needs’ Mental Health and Development Programme^55^ involved community volunteers and lay health workers to enhance the effectiveness of the programme.

Despite these similarities, some differences exist between initiatives in HICs and LMICs. HIC programmes, such as The Station Community Mental Health Centre,^66^ often operate within formal institutional frameworks, focussing on broader psychosocial outcomes like empowerment and stigma reduction. In contrast, LMIC programmes were often more grassroots in nature, relying heavily on trained lay workers and volunteers to deliver interventions resource-constrained settings. Examples include the Temple Healing initiative in India^62^ and the Friendship Bench in Zimbabwe,^45,52^ which integrated local cultural practices and traditions to enhance community acceptability, a feature less commonly seen in HICs. Indeed, initiatives in LMICs were more likely to prioritise cultural relevance as exemplified in various programmes.^39,45,50,52,61,64^ Additionally, while HIC initiatives emphasised formal collaboration and structured delivery, LMIC programmes tended to prioritise direct clinical recovery and wellbeing outcomes, such as symptom reduction and improved quality of life, and the sustainability of initiatives were more likely to be affected by funding in LMIC.^52^

## Discussion

This is the first scoping review to map the available literature on community-engaged mental health and wellbeing initiatives in under-resourced settings in LMIC and HIC. According to the WHO^19^ definition, community engagement involves a continuum of community involvement in the design and implementation of initiatives: from low-level approaches where community are informed, to mid-level where community members are consulted, and to high-level approaches including collaboration and decision-making with community members, or community-led approaches.

Our review of 35 studies, comprising 29 mental health initiatives, indicates that community-engaged approaches are frequently well received by the target population and can positively impact clinical recovery outcomes (eg, reduction in mental health symptoms), wellbeing (eg, QoL), personal recovery indicators (eg, improved social and family connection, meaningful occupation, self-efficacy and acceptance, and empowerment), and access to care. Those with higher levels of engagement, and particularly community-led initiatives, were more consistently reported as positive by community members who were delivering or receiving the intervention. Nonetheless, implementation efforts could be hampered by the perceived acceptability and accessibility of initiatives. For example, mental health training or mental health interventions could be perceived as irrelevant and or unacceptable,^40,50,58^ and physical distance, poor knowledge, or referral process could impact on accessibility.^51,52,58^ Other barriers included overburdening of community members and community mistrust and unwillingness to share resources.^43^

Reported barriers may reflect the lack of community engagement in design of initiatives. Although mid-level and high-level engagement was reported, it was only in the implementation (eg, screening, referrals, and delivery of mental health interventions), but not the design stages. As Arnstein^69^ originally argued, lower-levels of engagement in the design stages gives communities ‘little opportunity to influence’ development of a programme or initiative (p. 219). Our study indicates that a lack of community influence in the design of mental health initiatives may limit the perceived acceptability and accessibility, and lead to mistrust and unwillingness of community partners to share community assets. Hawke et al^70^ argue for involvement of community members in the design of mental health interventions and programmes from inception, through to implementation, and evaluation, to improve the perceived relevance and accessibility to the target population. Grindell et al^71^ note that ‘co’ approaches, where community members are equal partners in the design of initiatives, not only creates more relevant and acceptable approaches, but also instils a sense of community ownership, trust, and confidence in healthcare solutions. This is particularly important as our study found that building community trust was a key facilitator of programme success.^35^ Nonetheless, our findings indicate that engaging with community at the inception of a programme was more effective for promoting cultural safety and community acceptance,^35^ and the need for acceptable and culturally relevant interventions could drive higher levels of community engagement across the lifespan of a programme.^41^

Programmes incorporating community members typically involved community ‘peers’, including village leaders, religious leaders or groups, Indigenous people, or pregnant women and mothers. However, despite emphasis internationally on meaningful engagement of people with lived experience of mental health challenges in health initiatives,^21^ and incorporation of peer workers with a lived experience of mental challenges into mental health service delivery,^72^ none of the included studies indicated people with lived experience of mental health challenges as community peers. Perhaps lack of involvement is reflective of the perceived high stakes of tackling mental health challenges in under-resourced settings. Fran Baum^73^ notes, that the ‘bigger the stakes’, the more chance that marginalised community members will be excluded from participatory processes (p. 534).

However, the exclusion of people with lived experience in community-engagement initiatives is likely linked to entrenched and systemic prejudice towards people with mental health challenges, who are frequently excluded from health promotion initiatives.^15,74^ Indeed, studies in our review reported that mental health initiatives could be undermined by community workers’ and community members’ stigma towards people with mental health challenges, who they perceived as lacking capacity to make autonomous choices and were deemed unfit to socialise with or live in proximity to.^39,63^ Arguably, the involvement of people with lived experience of mental health challenges in the design and delivery of mental health initiatives in under-resourced settings might support reduction of stigma and discrimination towards this population. It was the case that women community volunteers in the Atmiyata programme played a vital role in addressing gender and caste barriers.^50^

Perhaps the value of involving people with lived experience in mental health initiatives cannot be overstated. In addition to enhancing inclusion and justice for people with lived experience,^74^ research indicates that the involvement of people with lived experience of mental health challenges in the design and delivery of mental health interventions and programmes also improves the perceived relevance and impact, as well as enhancing sustainability.^70^ However, no included studies used high-level engagement approaches with people with lived experience of mental health challenges through all stages of design and delivery, which may have the potential to further enhance mental health and wellbeing initiatives, particularly those that come up against issues of entrenched mental health stigma. Additionally, given the heterogeneity of communities, and the intersectional nature of health inequity, meaningful engagement in mental health interventions arguably requires careful involvement of people with lived experience of mental health challenges, as well as those with a lived experience of social inequities, for example, related to gender, ethnicity, social class etc.^15^

Beyond aspects of community involvement, our findings indicate that primary health doctors could be unwilling to prescribe medications due to a lack of training,^50^ and community health workers could be unwilling to collaborate in care, and rejected the choices of people with diagnosed mental health challenges.^39^ Further training of healthcare workers is required to ensure that integrated mental health services are willing and able to take referrals, and to provide treatment and care that is effective and aligns with the needs and requests of people with lived experience. Additionally, as Baum^73^ notes, involvement of health workers in the design and delivery of initiatives and programmes – working alongside community members and people with lived experience – may support the development of motivation and trust to participate in community-engaged initiatives, as well as respect for community self-determination and healthcare priorities of people with lived experience.

### Review Limitations

Despite the strengths of this review for examining community-engaged mental health and wellbeing initiatives from both LMIC and HIC, the findings should be interpreted considering its limitations. First, it is important to note that the programmes’ impact was not a cause-effect relationship but associations between the initiatives and various aspects of mental health or wellbeing. As noted in one of the included studies,^51^ researchers indicated uncertainty about the effectiveness of programmes due to uncontrolled variables, including changes in the broader social determinants of health within communities over time. Second, even though community members were involved, the involvement of people with lived experiences of mental health challenges was not evident in the design or implementation of the initiatives. Third, some initiatives involving high community engagement/leadership were assessed through pilot studies (eg, Chomat et al^46^ and Brown et al^42^) and included in this review. It remains uncertain if these programmes would remain impactful with large-scale implementation.

Additionally, caution is warranted when interpreting findings, due to the potential for positive reporting and publication biases, particularly in community-led initiatives, and from studies with below-average methodological quality.^43,56^ It is important to note that this study adopts a scoping review approach to address the lack of prior comprehensive reviews on this topic. Nevertheless, methodological elements typically associated with systematic reviews, such as quality ratings of included studies, were incorporated to enhance transparency in reporting, and rigour and credibility of the review.

## Conclusion

This review highlighted the critical role of community-engaged initiatives in promoting mental health and well-being of communities in under-resourced settings. The initiatives targeted peer programmes, particularly in LMIC (eg, involving laypeople and religious leaders), collaborative care approaches (in collaboration with primary healthcare), and integrative services (beyond mental health eg, physical health). These programmes underscore the diversity of various community-based care initiatives across different populations/income settings. The findings reveal that actively engaging community members in the design, implementation, or leadership of community initiatives generally led to positive outcomes in various mental health and broader well-being measures. In particular, programmes with high community engagement, including leadership, could be instrumental in reducing clinical mental health symptoms, promoting personal recovery (eg, social connection, empowerment, and meaningful occupation), and improving mental health literacy and access to care/services. However, it is unclear if these benefits were sustained over long periods of time, and there were mixed results on quality of life and the impact of initiatives on individuals diagnosed with mental health conditions such as psychosis and schizophrenia.

While most studies demonstrated positive outcomes, there were various context-specific challenges, including limited resources, accessibility, and cultural barriers, which necessitated adaptations to ensure programme relevance and acceptance. The need for culturally tailored approaches was particularly evident in diverse populations such as Indian rural area residents and Australian Indigenous communities. None of the studies indicated high-level involvement of community members in design of mental health initiatives, or involvement of people with lived experience of mental health challenges. Leveraging local knowledge, fostering active community leadership, and involving community members with intersecting experience of mental health challenges and social inequity in the design, delivery, and evaluation of mental health and wellbeing initiatives, could achieve improved outcomes of underserved populations of high-income countries as well as LMIC. Future studies should focus on scalable initiatives, long-term impact, and inclusion of people with lived experience of mental health challenges and social inequities in programme design, implementation and evaluation.

## Supplemental Material

sj-docx-1-jpc-10.1177_21501319251332723 – Supplemental material for Community-Engaged Mental Health and Wellbeing Initiatives in Under-Resourced Settings: A Scoping Review of Primary Studies Supplemental material, sj-docx-1-jpc-10.1177_21501319251332723 for Community-Engaged Mental Health and Wellbeing Initiatives in Under-Resourced Settings: A Scoping Review of Primary Studies by Muhammad Chutiyami, Natalie Cutler, Sopin Sangon, Tusana Thaweekoon, Patcharin Nintachan, Wilai Napa, Phachongchit Kraithaworn and Jo River in Journal of Primary Care & Community Health
