# Supplementary material for: Community-Engaged Mental Health and Wellbeing Initiatives in Under-Resourced Settings: A Scoping Review of Primary Studies
Source: J Prim Care Community Health. 2025 Apr 21;16:21501319251332723. doi: 10.1177/21501319251332723 (PMC12035253; doi:10.1177/21501319251332723)
Supplement: sj-docx-1-jpc-10.1177_21501319251332723 – Supplemental material for Community-Engaged Mental Health and Wellbeing Initiatives in Under-Resourced Settings: A Scoping Review of Primary Studies [file sj-docx-1-jpc-10.1177_21501319251332723.docx]

## Community-engaged mental health and wellbeing initiatives in under-resourced settings: a scoping review of primary studies

**Supplemental table 1: Search terms**

| Database | Search Query strings |
| --- | --- |
| Ovid MEDLINE | (disadvantag* or remote or "socially deprived" or underserve* or "low and middle income" or lmic or poverty) AND (Rural Population or Poverty or Poverty Areas) AND (Community Participation or Community Support) AND (communit* adj3 (led or develop or outreach or involve* or engage* or based or intervention or model* or program*) AND (mental health or wellbeing or well being).tw. |
| Embase | (disadvantag* or remote or "socially deprived" or underserve* or "low and middle income" or lmic or poverty).tw. AND (rural population or poverty) AND (community participation or community support or communit* adj3 (led or develop or outreach or involve* or engage* or based or intervention or model* or program*)).tw. AND (mental health or wellbeing or well being).tw. |
| Scopus | (disadvantag* or remote or "socially deprived" or underserve* or "low and middle income" or lmic or poverty)  AND (communit* W/3 (led or develop or outreach or involve* or engage* or based or intervention or model* or program*))  AND ("mental health" or wellbeing or "well being") |
| EBSCOhost- CINAHL | ( (disadvantag* or remote or "socially deprived" or underserve* or "low and middle income" or lmic or poverty) ) OR AB ( (disadvantag* or remote or "socially deprived" or underserve* or "low and middle income" or lmic or poverty) ) AND (MH "Rural Population") AND (MH "Poverty") OR (MH "Poverty Areas") AND (MH "Community Support") AND TI ( (communit* N3 (led or develop or outreach or involve* or engage* or based or intervention or model* or program*)) ) OR AB ( (communit* N3 (led or develop or outreach or involve* or engage* or based or intervention or model* or program*)) ) AND (MH "Mental Health") AND TI ( (mental health or wellbeing or well being) ) OR AB ( (mental health or wellbeing or well being) ) |
| EBSCOhost-PsycINFO | TI ( (disadvantag* or remote or "socially deprived" or underserve* or "low and middle income" or lmic or poverty) ) OR AB ( (disadvantag* or remote or "socially deprived" or underserve* or "low and middle income" or lmic or poverty) ) OR ( (Rural population) ) OR (DE "Poverty" OR DE "Poverty Areas") AND (TI ( (communit* N3 (led or develop or outreach or involve* or engage* or based or intervention or model* or program* or participation)) ) OR AB ( (communit* N3 (led or develop or outreach or involve* or engage* or based or intervention or model* or program*)) ) AND DE "Community Involvement" AND TI ( (mental health or wellbeing or well being) ) OR AB ( (mental health or wellbeing or well being) ) AND DE "Mental Health" |

**Supplemental Table 2: Quality Appraisal of Included Studies**

1. **JBI for Cross-sectional studies/surveys**

| Study | 1 | 2 | 3 | 4 | 5 | 6 | 7 | 8 | Total Y |
| --- | --- | --- | --- | --- | --- | --- | --- | --- | --- |
| Anwar-McHenry, et al ^37^ | U | Y | Y | Y | Y | Y | Y | Y | 7/8 |
| Chung, et al ^51^ | U | Y | U | Y | Y | Y | Y | Y | 6/8 |

*Key:* Y, yes; N, no; U, unclear; NA, not applicable. *Total:* Computed by summing the number of ‘yes (Y)’ scores obtained

*Questions:*

1. Were the criteria for inclusion in the sample clearly defined?

2. Were the study subjects and the setting described in detail?

3. Was the exposure measured validly and reliably?

4. Were objective, standard criteria used for measurement of the condition?

5. Were confounding factors identified?

6. Were strategies to deal with confounding factors stated?

7. Were the outcomes measured validly and reliably?

8. Was appropriate statistical analysis used?

Further information at <https://jbi-global-wiki.refined.site/space/MANUAL/4689081/Appendix+7.5+Critical+appraisal+checklist+for+analytical+cross-sectional+studies>

1. **JBI for Qualitative study**

| **Study** | **1** | **2** | **3** | **4** | **5** | **6** | **7** | **8** | **9** | **10** | **Total** |
| --- | --- | --- | --- | --- | --- | --- | --- | --- | --- | --- | --- |
| Appiah et al ^59^ | Y | Y | Y | Y | Y | Y | Y | Y | Y | Y | 10/10 |
| Asher et al ^60^ | Y | Y | Y | Y | Y | Y | U | Y | Y | Y | 9/10 |
| Bryant, et al ^44^ | Y | Y | Y | U | U | U | NA | NA | U | Y | 4/10 |
| Giusto, et al ^62^ | Y | Y | Y | Y | Y | Y | Y | Y | Y | Y | 10/10 |
| Kermode, et al ^52^ | Y | Y | Y | Y | Y | U | U | Y | Y | Y | 8/10 |
| Nasir, et al ^42^ | Y | Y | Y | Y | Y | Y | U | Y | U | Y | 8/10 |
| Taylor, et al ^68^ | Y | Y | Y | Y | Y | U | NA | Y | Y | Y | 8/10 |
| van Ginneken, et al ^40^ | U | Y | Y | Y | Y | NA | NA | U | Y | Y | 6/10 |
| Kidia et al ^67^ | U | Y | Y | Y | Y | Y | Y | Y | Y | Y | 9/10 |

*Key:* Y, yes; N, no; U, unclear; NA, not applicable.

1. Is there congruity between the stated philosophical perspective and the research methodology?

2. Is there congruity between the research methodology and the research question or objectives?

3. Is there congruity between the research methodology and the methods used to collect data?

4. Is there congruity between the research methodology and the representation and analysis of data?

5. Is there congruity between the research methodology and the interpretation of results?

6. Is there a statement locating the researcher culturally or theoretically?

7. Is the influence of the researcher on the research, and vice- versa, addressed?

8. Are participants, and their voices, adequately represented?

9. Is the research ethical according to current criteria or, for recent studies, and is there evidence of ethical approval by an appropriate body?

10. Do the conclusions drawn in the research report flow from the analysis, or interpretation, of the data?

Further information at <https://jbi-global-wiki.refined.site/space/MANUAL/4687851/Appendix+2.2%3A+Discussion+of+JBI+Qualitative+critical+appraisal+criteria>

1. **JBI case series/case study**

| **Study** | **1** | **2** | **3** | **4** | **5** | **6** | **7** | **8** | **9** | **10** | **Total** |
| --- | --- | --- | --- | --- | --- | --- | --- | --- | --- | --- | --- |
| Shields‑Zeeman, et al ^66^ | Y | Y | Y | U | Y | Y | U | N | Y | U | 6/10 |
| Jayaram et al ^33^ | Y | U | Y | Y | Y | Y | Y | U | Y | NA | 7/10 |

*Key:* Y, yes; N, no; U, unclear; NA, not applicable

1. Were there clear criteria for inclusion in the case series?
2. Was the condition measured in a standard, reliable way for all participants included in the case series?
3. Were valid methods used for identification of the condition for all participants included in the case series?
4. Did the case series have consecutive inclusion of participants?
5. Did the case series have complete inclusion of participants?
6. Was there clear reporting of the demographics of the participants in the study?
7. Was there clear reporting of clinical information of the participants?
8. Were the outcomes or follow up results of cases clearly reported?
9. Was there clear reporting of the presenting site(s)/clinic(s) demographic information?
10. Was statistical analysis appropriate?

Further information at <https://jbi-global-wiki.refined.site/space/MANUAL/4689063/Appendix+7.3+Critical+appraisal+checklists+for+case+series>

1. **JBI for Cohort study**

| **Study** | **1** | **2** | **3** | **4** | **5** | **6** | **7** | **8** | **9** | **10** | **11** | **Total** |
| --- | --- | --- | --- | --- | --- | --- | --- | --- | --- | --- | --- | --- |
| Lund, et al ^64^ | N | N | Y | Y | Y | Y | Y | Y | Y | Y | Y | 8/11 |

*Key:* Y, yes; N, no; U, unclear; NA, not applicable.

1. Were the two groups similar and recruited from the same population?
2. Were the exposures measured similarly to assign people to both exposed and unexposed groups?
3. Was the exposure measured in a valid and reliable way?
4. Were confounding factors identified?
5. Were strategies to deal with confounding factors stated?
6. Were the groups/participants free of the outcome at the start of the study (or at the moment of exposure)?
7. Were the outcomes measured in a valid and reliable way?
8. Was the follow up time reported and sufficient to be long enough for outcomes to occur?
9. Was follow up complete, and if not, were the reasons to loss to follow up described and explored?
10. Were strategies to address incomplete follow up utilized?
11. Was appropriate statistical analysis used?

Further information at <https://jbi-global-wiki.refined.site/space/MANUAL/4689113/Appendix+7.1++Critical+appraisal+checklist+for+cohort+studies>

1. **JBI for RCTs**

| **Study** | **1** | **2** | **3** | **4** | **5** | **6** | **7** | **8** | **9** | **10** | **11** | **12** | **13** | **Total** |
| --- | --- | --- | --- | --- | --- | --- | --- | --- | --- | --- | --- | --- | --- | --- |
| Pathare et al ^58^ | Y | Y | Y | U | U | Y | U | Y | Y | Y | Y | Y | Y | 10/13 |

*Key:* Y, yes; N, no; U, unclear; NA, not applicable.

1. Was true randomization used for the assignment of participants to treatment groups?
2. Was allocation to treatment groups concealed?
3. Were treatment groups similar at the baseline?
4. Were participants blind to treatment assignment?
5. Were those delivering treatment blind to treatment assignment?
6. Were outcomes assessors blind to treatment assignment?
7. Were treatment groups treated identically other than the intervention of interest?
8. Was follow-up complete and if not, were differences between groups in terms of their follow up adequately described and analyzed?
9. Were participants analyzed in the groups to which they were randomized?
10. Were outcomes measured in the same way for treatment groups?
11. Were outcomes measured in a reliable way?
12. Was appropriate statistical analysis used?
13. Was the trial design appropriate, and any deviations from the standard RCT design (individual randomization, parallel groups) accounted for in the conduct and analysis of the trial?

Further information for JBI RCT at <https://jbi-global-wiki.refined.site/space/MANUAL/4689894/Appendix+3.2%3A+Discussion+of+JBI+appraisal+criteria+for+randomized+controlled+trials>

1. **JBI for non-RCT Study**

| **Study** | **1** | **2** | **3** | **4** | **5** | **6** | **7** | **8** | **9** | **total** |
| --- | --- | --- | --- | --- | --- | --- | --- | --- | --- | --- |
| Aréchiga, et al ^50^ | Y | Y | Y | N | Y | NA | Y | Y | Y | 7/9 |
| Brown, et al ^35^ | Y | Y | Y | N | Y | NA | Y | Y | Y | 7/9 |
| Capp, et al ^41^ | Y | Y | Y | N | Y | NA | Y | Y | Y | 7/9 |
| Chibanda, et al ^56^ | Y | Y | NA | N | Y | Y | Y | Y | Y | 7/9 |
| Lam, et al ^46^ | Y | Y | Y | Y | Y | Y | Y | Y | Y | 9/9 |
| Raguram, et al ^57^ | Y | Y | Y | N | N | NA | Y | Y | Y | 6/9 |
| Shidhaye, et al ^55^ | Y | Y | Y | N | Y | Y | NA | Y | Y | 7/9 |
| Sun, et al ^39^ | Y | Y | NA | N | Y | NA | Y | Y | Y | 6/9 |

*Key:* Y, yes; N, no; U, unclear; NA, not applicable

1. Is it clear in the study what is the ‘cause’ and what is the ‘effect’ (i.e. there is no confusion about which variable comes first)?
2. Were the participants included in any comparisons similar?
3. Were the participants included in any comparisons receiving similar treatment/care, other than the exposure or intervention of interest?
4. Was there a control group?
5. Were there multiple measurements of the outcome both pre and post the intervention/exposure?
6. Was follow up complete and if not, were differences between groups in terms of their follow up adequately described and analyzed?
7. Were the outcomes of participants included in any comparisons measured in the same way?
8. Were outcomes measured in a reliable way?
9. Was appropriate statistical analysis used?

Further information at <https://jbi-global-wiki.refined.site/space/MANUAL/4689854/Appendix+3.4%3A+Discussion+of+JBI+appraisal+criteria+for+Quasi-Experimental+Studies+(non-randomized+experimental+studies)>

1. **Mixed method Study**

Quality appraisal for Mixed-Method Studies

| **Study** | **1** | **2** | **3** | **4** | **5** | **Total** |
| --- | --- | --- | --- | --- | --- | --- |
| Ali, et al ^43^ | Y | Y | Y | U | Y | 4/5 |
| Asher, et al ^34^ | Y | Y | Y | Y | Y | 5/5 |
| Asher, et al ^61^ | Y | Y | Y | Y | Y | 5/5 |
| Balaji, et al ^65^ | Y | Y | Y | N | N | 3/5 |
| Chomat, et al ^49^ | Y | Y | Y | Y | Y | 5/5 |
| Iheannacho, et al ^63^ | Y | Y | Y | U | Y | 4/5 |
| Joag et al ^45^ | Y | Y | Y | Y | Y | 5/5 |
| Lee et al ^47^ | Y | Y | Y | Y | Y | 5/5 |
| Mathias et al ^53^ | Y | Y | N | N | N | 2/5 |
| Mendel, et al ^48^ | Y | Y | Y | Y | Y | 5/5 |
| Nguyen et al ^54^ | Y | Y | Y | Y | Y | 5/5 |
| Nickels et al ^38^ | Y | Y | Y | Y | Y | 5/5 |

Key: Y=yes, N=no, U=unclear/can’t tell

1. Is there an adequate rationale for using a mixed methods design to address the research question?
2. Are the different components of the study effectively integrated to answer the research question?
3. Are the outputs of the integration of qualitative and quantitative components adequately interpreted?
4. Are divergences and inconsistencies between quantitative and qualitative results adequately addressed?
5. Do the different components of the study adhere to the quality criteria of each tradition of the methods involved?

Further information at <http://mixedmethodsappraisaltoolpublic.pbworks.com/w/page/127425845/Download%20the%20MMAT>
